# Abundance of fecal indicator bacteria and diversity of *Escherichia coli* associated with poultry farms and pasture land cover in streams of northwestern South Carolina

**DOI:** 10.1007/s10661-024-13499-w

**Published:** 2024-12-04

**Authors:** Virginia H. Britt, Min-Ken Liao, Gregory P. Lewis

**Affiliations:** Department of Biology, Furman University, Greenville, SC USA

**Keywords:** Fecal indicator bacteria, *Escherichia coli*, Streams, Land cover/land use, Pasture, Poultry

## Abstract

Livestock can contribute fecal indicator bacteria (FIB) to waterbodies. However, few studies have examined the relationship between watershed land use or land cover involving livestock production and the genetic diversity of *Escherichia coli* in freshwater ecosystems. Our major goal was to determine if FIB abundance and *E. coli* phylogenetic group distributions in headwater streams are related to livestock production in rural watersheds in South Carolina. In both 2017 and 2018, grab samples were collected from streams at summer baseflow. In 2017, we collected samples from watersheds with or without poultry rearing facilities (PRFs). In 2018, we collected samples from streams draining watersheds with mixed forest and pasture cover and from streams in mostly forested watersheds. In both summers, we measured concentrations of total coliforms, *E. coli*, and *Enterococcus*. We also categorized *E. coli* isolates into one of four phylogenetic groups (A, B1, B2, D). Streams with PRFs in their watersheds had significantly higher concentrations of *Enterococcus* but not total coliforms or *E. coli* than streams in watersheds without PRFs. Also, B2 isolates were less frequent and B1 isolates were more frequent in watersheds with PRFs than in those without. Streams draining mixed forest/pasture watersheds had significantly higher concentrations of total coliforms and *E. coli* but not *Enterococcus*, as well as higher frequencies of B1 isolates, than streams in mostly forested watersheds. Overall, the most frequent *E. coli* phylogenetic groups in watersheds with animal production appeared consistent with the phylogenetic groups that are especially abundant in poultry or mammalian livestock feces.

**Supplementary Information:**

The online version contains supplementary material available at 10.1007/s10661-024-13499-w.

## Introduction

Many studies have shown that animal production systems can influence the abundance of fecal indicator bacteria (FIB) in surface waters (e.g., Baxter-Potter & Gilliland, 1988; Bradshaw et al., 2016; Crowther et al., 2002; Mallin et al., 2015). For example, it has been shown that bacteria from manure deposited onto grazed pasture or used as crop fertilizer can travel into waterways by means of surface runoff or groundwater transport (Harmel et al., 2013; Hruby et al., 2016; Hubbard et al., 2020). Additional studies of cattle manure and poultry litter deposited on pastures (Avery et al., 2004; Garzio-Hadzick et al., 2010; Harmel et al., 2013) or used as crop fertilizer (Islam et al., 2004) show that FIB have the potential to survive in soil and subsequently contaminate water sources via surface runoff (Avery et al., 2004; Lau & Ingham, 2001; Stocker et al., 2015). In addition, cattle may be sources of FIB to streams or rivers where the animals have direct access to channels (e.g., Davies-Colley et al., 2004).

Although it is clear that animal production systems contribute FIB to surface waters, it is less well understood to what extent animal production systems influence the genetic diversity of FIB in surface waters. A good model organism for such studies is *Escherichia coli (E. coli)*, a Gram-negative inhabitant of the intestines and feces of endothermic animals (Tenaillon et al., 2010). Although most strains of *E. coli* are non-pathogenic commensals, some strains cause disease in humans and domesticated animals (Kuhnert et al., 2000). Previous studies have used a variety of methods for determining the diversity of strains in both animal and environmental samples (e.g., Cook et al., 2011; McLellan, 2004; Wijesinghe et al., 2009). One method of classifying strains of *E. coli* is the use of the reference collection (ECOR) of Gordon et al. (2008). Most (>85%) strains of *E. coli* fall into one of four phylogroups: A, B1, B2, and D (Gordon et al., 2008). These four phylogroups are linked to certain phenotypic characteristics and may fill different ecological niches. Groups A and B1 are thought to be sister clades divergent from the ancestral groups B2 and D (Salipante et al. 2015). Although every phylogroup contains pathogenic strains, group B2 and, to a lesser extent, group D strains tend to have the highest number of potential virulence factors (Cunha et al. 2014; Johnson et al. 2002; Moreno et al. 2009; Salipante et al. 2015). Group A and B1 strains, on the other hand, are more likely to be commensal (Bailey et al. 2010). Additionally, host diet, host body mass, climate, and other factors can influence the distribution of *E. coli* types among host species, both wild and domestic (Escobar-Páramo et al., 2006; Gordon & Cowling, 2003). Because only a few truly host-specific strains of *E. coli* have been identified, Carlos et al. (2010) suggest that an effective approach for monitoring sources of fecal contamination would be to study and compare *E. coli* population genetic structures in different hosts and environments. On the other hand, selection for certain strains by the soil or water environment after deposition in animal feces may reduce any “signature” of domesticated animal sources (Jang et al., 2017).

Although many studies have examined distributions of *E. coli* phylogroups in human and animal feces (e.g., Carlos et al., 2010; Escobar-Páramo et al., 2006; Lagerstrom & Hadly, 2023; Unno et al., 2009) and in soils (e.g., Dusek et al., 2018; NandaKafle et al., 2017), few studies have examined phylogroup distributions in surface waters. Higgins et al. (2007) conducted preliminary analyses of phylogroup distributions from one urban stream, one agricultural stream, and “rural surface waters” in Maryland. Cook et al. (2011) examined phylogroup distributions in a single stream in an agricultural area of western Kentucky as well as in manure from swine, poultry, and dairy cattle. Tymensen et al. (2015) examined phylogroup distributions at four sites along the Milk River in Alberta, Canada, which drains a watershed with predominantly grassland cover and which included some cattle production. Although these studies collected samples over several months’ time and/or under varying flow conditions, they were limited in the geographic scope of their sampling and were not designed to test whether phylogroup distributions differed significantly among watersheds with different land covers, land uses, or animal production systems.

Our major goal in this study was to determine if FIB abundance and *E. coli* phylogroup distributions in headwater streams are related to animal production systems in the contributing watersheds. We conducted our study in northwestern South Carolina, mainly in the Piedmont physiographic province, where pasture is a widespread land cover and poultry-rearing facilities (PRFs) operate in some watersheds. Regarding animal production systems, we made two major comparisons. First, we compared streams in rural watersheds with and without PRFs. We hypothesized that the abundance of FIB would be higher in watersheds with PRFs than in those without, and that the phylogroup distributions within *E. coli* populations in watersheds with PRFs would differ from those without PRFs. Specifically, based on the results of prior studies of *E. coli* phylogroups in different animal hosts (e.g., Coura et al., 2015, 2017; Hiki et al., 2014; Unno et al., 2009), we expected to find higher proportions of phylogroups A and B1 in watersheds with PRFs. Because we aimed to isolate and classify at least 300 *E. coli* strains from the environment to facilitate comparisons within our study and potentially to previous studies, we chose to use the simpler and less expensive traditional Clermont phylotyping method (Clermont et al., 2000) rather than the newer method (Clermont et al., 2013). Second, we compared watersheds with mixtures of forest and pasture cover to watersheds with mostly forest cover. In this comparison, we hypothesized that FIB abundance would be higher in streams draining the mixed forest/pasture watersheds than in the mostly forested watersheds. Based on results of prior studies (e.g., Coura et al., 2015; Howard et al., 2017; Tymensen et al., 2015; Unno et al., 2009), we also hypothesized that mixed forest/pasture watersheds would have higher proportions of the phylogroup group B1, which is associated with cattle feces. Finally, by sampling some watersheds in two subsequent years, we examined year-to-year variation in FIB abundance and phylogroup distribution of *E. coli* populations.

## Materials and methods

### Study area and sample collection

**Fig. 1:**
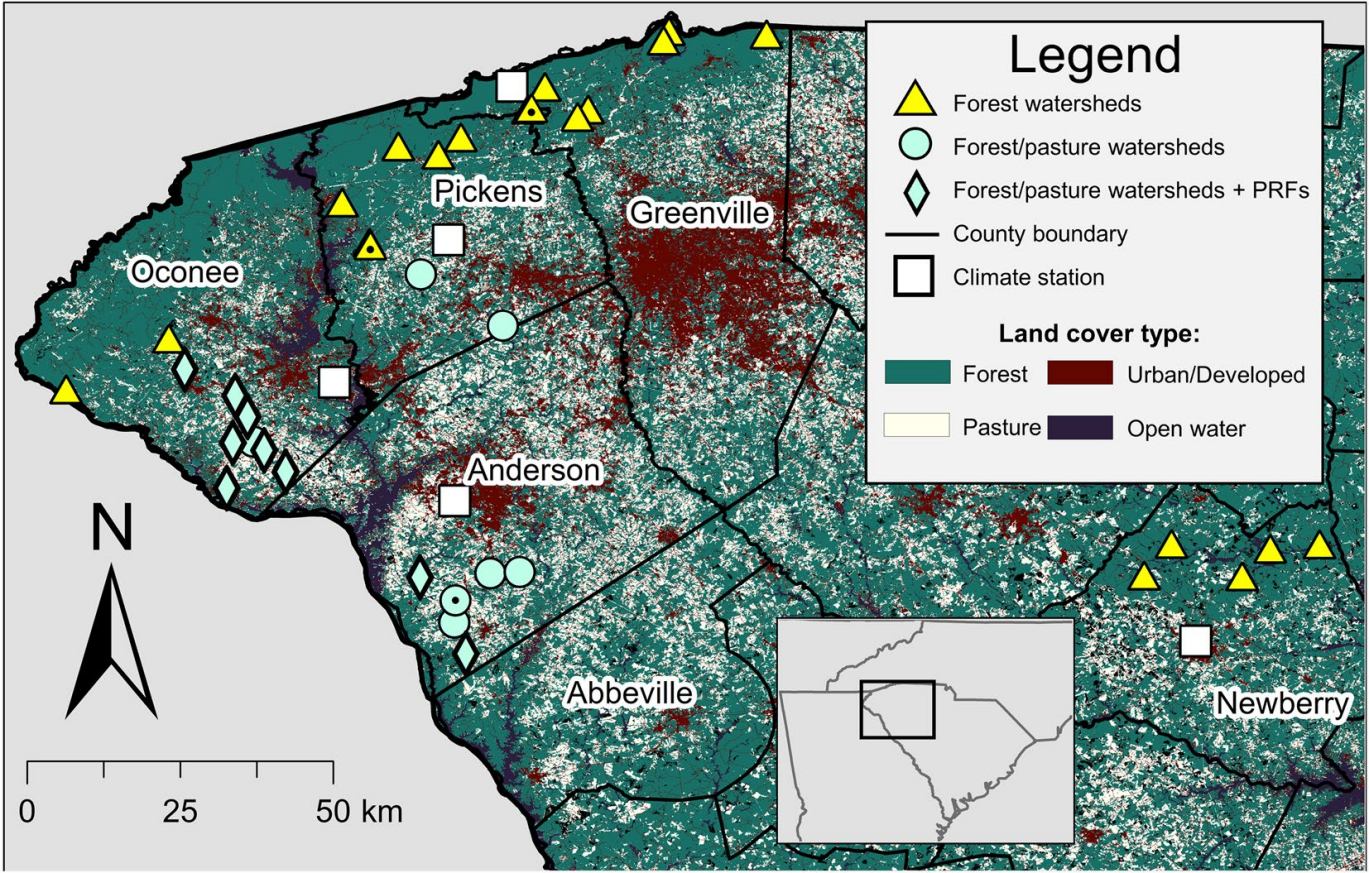
Locations of streams sampled in northwestern South Carolina. Each stream drained a watershed categorized as either a “Forest” watershed (>72% forest cover) or as a “Forest/pasture” watershed (>76% combined forest and grass/pasture land cover). Poultry rearing facilities (PRFs) occurred within nine of the “Forest/pasture” watersheds (“Forest/pasture watersheds + PRFs). “Pasture” land cover includes both the “Pasture/hay” and “Grassland/herbaceous” categories from the U. S. Geological Survey’s National Land Cover Database. Black dots within sample location symbols denote overlapping symbols for two sample sites that were located in close proximity to each other. Climate stations are the following stations from the US National Oceanic and Atmospheric Administration network: Anderson County (Anderson County Airport, station 93846), Greenville County (Caesars Head, station 381256), Newberry County (Newberry, station 386209), Oconee County (Clemson/Oconee County Airport, station 53850), and Pickens County (Pickens, station 386831)

We conducted our study within rural watersheds in the Piedmont and Blue Ridge Physiographic Provinces of northwestern South Carolina (Fig. 1). The climate of the region is warm temperate and varies with elevation. For example, based on 1981–2010 records, normal annual precipitation varies from ~122 cm at Newberry (elevation 145 m) to ~178 cm at Caesars Head (elevation 975 m) (National Oceanic and Atmospheric Administration (NOAA), 2020). Also, daily mean temperatures during the summer months (June–August) range from ~26.4 °C at Newberry to ~ 20.6 °C at Caesars Head (National Oceanic and Atmospheric Administration (NOAA), 2020).

In the study area, forest and pasture are the predominant rural land covers. Livestock on pastures were primarily cattle. According to the 2017 Census of Agriculture of the US Department of Agriculture (USDA), cattle accounted for 79%, 85%, and 64% of livestock by head in Anderson, Oconee, and Pickens Counties, respectively (United States Department of Agriculture (USDA), 2019). Horses accounted for about 7%, 6%, and 14% of livestock, and goats accounted for about 10%, 4%, and 11% of livestock in these counties (United States Department of Agriculture (USDA), 2019). Based on land cover data from the 2016 National Land Cover Database (NLCD) of the United States Geological Survey (accessed April 30, 2020, via the CropScape web application, https://nassgeodata.gmu.edu/CropScape/) and the 2017 USDA livestock census, total livestock densities at the county level on grass/pasture land in Anderson, Oconee, and Pickens Counties would have been about 112, 109, and 73 head/km^2^. Also, according to the 2017 USDA census, Oconee County had the second highest population of broiler chickens in South Carolina at about 7.2 million birds (~4470 birds/km^2^ of county land area). Anderson County had a population of about 2.2 million birds (~1190 birds/km^2^ of county land area), while Pickens County had a population of only about 840 birds (~0.7 birds/km^2^ of county land area) (United States Department of Agriculture (USDA), 2019).

Streams were selected initially based on visual assessment of watershed land cover and accessibility from roads using Google Earth imagery. Watershed Modeling Systems (Aquaveo, Provo, Utah, USA) versions 10.0 and 10.1 were used to delineate drainage areas upstream of each sampling site based on digital elevation model input. Land cover data from the US Geological Survey’s 2011 NLCD were accessed using the CropScape web application (https://nassgeodata.gmu.edu/CropScape/). Land cover percentages within each watershed were then determined using ArcMap 10.3.1 (Environmental Systems Research Institute, Redlands, California, USA). For some watersheds, separate “Pasture/hay” and “Grassland/herbaceous” land cover categories were obtained, whereas in others a single category of “Grassland/pasture” was obtained. For watersheds with the separate categories, “Pasture/hay” covered about 3 to 20 times more area than did “Grassland/herbaceous.” For categorizing watersheds and for making statistical comparisons, we combined the “Pasture/hay” and “Grassland/herbaceous” categories into a “Grass/pasture” category (Table 1). For watersheds in which PRFs occurred (Table 1), the densities of poultry houses in each of those watersheds (number of houses per unit area of watershed) were determined based on Google Earth imagery.

**Table 1:** Watershed and land cover data for stream sites sampled in 2017 and/or 2018 within the Blue Ridge and Piedmont provinces of northwestern South Carolina

Site	Drainage area (km^2^)	Sample site elevation (m)	Year(s) sampled*	Category	% forest	% grass/pasture	Poultry houses (number/km^2^)	County
BGR01	4.8	171	2017 (3)	Forest/pasture	47.7	38.6	0.8	Anderson
CFC01	6.0	260	2017 (1)	Forest/pasture	49.0	44.2	1.7	Oconee
CNO01	4.3	173	2017 (3)	Forest/pasture	20.1	62.6	1.4	Anderson
CRC01	14.2	237	2017 (4)	Forest/pasture	36.4	41.5	0.7	Oconee
FPC01	5.9	208	2017 (3)	Forest/pasture	39.1	45.7	1.4	Oconee
LCH01	4.3	250	2017 (3)	Forest/pasture	28.4	60.4	0.9	Oconee
MD02	8.2	246	2017 (4)	Forest/pasture	25.3	56.5	1.7	Oconee
SBD01	13.2	212	2017 (3)	Forest/pasture	26.9	50.1	3.6	Oconee
SNC02	3.1	256	2017 (3)	Forest/pasture	31.3	62.1	2.0	Oconee
CRM01	3.7	258	2017 (3), 2018 (1)	Forest/pasture	38.4	47.0	0	Pickens
LGC02	4.8	198	2017 (4), 2018 (1)	Forest/pasture	19.9	61.3	0	Anderson
LGC03	4.7	199	2017 (3), 2018 (1)	Forest/pasture	22.3	54.4	0	Anderson
LGC04	3.0	186	2017 (3), 2018 (1)	Forest/pasture	26.7	64.1	0	Anderson
MD03**	1.8	256	2017 (3), 2018 (1)	Forest/pasture	36.9	51.1	0	Oconee
TUG01	6.3	189	2017 (3), 2018 (2)	Forest/pasture	48.0	36.3	0	Anderson
TWV01	2.2	283	2017 (3), 2018 (1)	Forest/pasture	25.8	58.8	0	Pickens
WIC01	7.5	202	2017 (3), 2018 (2)	Forest/pasture	40.4	37.3	0	Anderson
BAR01	4.0	211	2018 (2)	Forest	98.4	1.4	0	Oconee
CRC03	3.0	287	2018 (2)	Forest	85.8	7.7	0	Oconee
IR05	7.8	118	2018 (2)	Forest	94.5	0.5	0	Newberry
IR10	16.1	119	2018 (2)	Forest	89.6	3.1	0.3	Newberry
KC02	8.2	97	2018 (2)	Forest	80.4	9.3	0.6	Newberry
LCC01	5.5	254	2018 (3)	Forest	90.4	1.4	0	Pickens
LCC02	3.2	254	2018 (2)	Forest	77.4	16.6	0	Pickens
POC01	2.9	292	2018 (3)	Forest	96.1	0.2	0	Pickens
REC01	6.6	579	2018 (2)	Forest	99.1	0.1	0	Pickens
SC01	9.4	87	2018 (2)	Forest	82.9	10.4	0.7	Newberry
UC01	4.9	88	2018 (2)	Forest	80.4	9.9	0	Newberry
US15A	7.2	322	2018 (2)	Forest	72.9	19.9	0	Greenville
US22	4.6	515	2018 (2)	Forest	96.0	0	0	Greenville
US23	5.6	590	2018 (2)	Forest	96.7	0	0	Greenville
US32	6.5	380	2018 (2)	Forest	94.6	0.7	0	Greenville
US60	3.4	369	2018 (2)	Forest	94.7	1.6	0	Pickens
US64	1.1	335	2018 (2)	Forest	92.0	1.0	0	Greenville
US65	1.7	323	2018 (2)	Forest	92.5	0	0	Greenville
US73	3.1	324	2018 (2)	Forest	97.7	1.5	0	Pickens
US79	1.6	307	2018 (2)	Forest	97.9	1.3	0	Greenville
VAC08	11.9	329	2018 (2)	Forest	96.0	1.0	0	Greenville

^*^The number of dates on which field measurements were made and water for laboratory analyses was collected within each year at each stream site is given in parentheses

^**^Located ~ 2.4 km upstream of MD02

During June 8–July 26, 2017, water samples from 17 second- and third-order streams draining watersheds in the upper Savannah River basin were collected (Table 1). Land cover in these watersheds was predominantly a mixture of forest and grass/pasture cover (Table 1). Total land cover in the “Developed/Low Intensity”, “Developed/Medium Intensity”, and “Developed/High Intensity” 2011 NLCD categories was <10% in all watersheds. Poultry-rearing facilities occurred within the watersheds of nine of these streams (Fig. 1, Table 1). Based on Google Earth imagery, distances from the stream sampling site to the nearest PRF in each watershed (stream channel length plus the shortest distance from the edge of the PRF to the stream channel) ranged from ~0.3 to ~2.3 km (mean = ~1.3 km).

During June 7–July 31, 2018, water samples from 29 first-, second-, and third-order streams draining watersheds in the Savannah, Saluda, and Broad River basins were collected (Table 1). These 29 streams included all eight sites from 2017 which drained watersheds with mixed pasture and forest land cover and which had no PRFs upstream (Table 1). Twenty-one additional sites were sampled in watersheds with predominantly (>70%) forested land cover (Fig. 1, Table 1). These sites were selected as part of a broader study of variation in water quality in forested watersheds across an elevation gradient in northwestern South Carolina (G. Lewis, unpublished). Poultry rearing facilities were found in the headwaters of three of these forested sites (Table 1). Distances from sampling site to the PRF in each watershed (stream channel length plus the shortest distance from the edge of the PRF to the stream channel) ranged from ~4.3 to ~5.2 km (mean = ~4.6 km) based on Google Earth imagery. Total land cover in the “Developed/Low Intensity”, “Developed/Medium Intensity”, and “Developed/High Intensity” 2011 NLCD categories was <8% in all 29 watersheds.

Precipitation amounts varied spatially and temporally during the sampling period (Supplementary Fig. 1). Because concentrations of FIB can vary widely along the rising and falling limbs of storm hydrographs in streams (e.g., Davies-Colley et al., 2008; McKergow & Davies-Colley, 2010), all water samples in both years were collected under baseflow conditions to minimize influence of surface runoff from precipitation events.

Each stream was sampled at the same location one to four times during a given summer (Table 1). For the eight streams sampled in both 2017 and 2018, we used the same location on each stream in both years. We sampled a larger number of streams over a larger geographic area in 2018 compared to 2017 (Table 1, Fig. 1). Due to resulting time constraints in 2018, we collected samples on fewer dates from each stream in 2018 than we did in 2017.

On each sampling date at each sampling location, pH, water temperature, specific conductivity, and dissolved oxygen were measured using a Fisher Scientific Accumet AP62 pH meter and a YSI 85 dissolved oxygen and conductivity meter. Stream discharge was estimated using a Swoffer Model 3000 current velocity meter and measurements of channel cross-sectional area. Water for bacterial analyses was collected in a sterile polypropylene bottle. Additional water for turbidity analysis was collected independently in a high-density polyethylene bottle. All water bottles were transported to the laboratory in the dark and on ice. Turbidity was measured upon return to the laboratory using a LaMotte 2020 turbidity meter. Bacterial analyses also were begun on the same day as water collection.

### Quantification of fecal indicator bacteria

Concentrations of total coliforms, *E. coli*, and *Enteroccus* spp. were measured using the IDEXX Colilert® and Enterolert® Quanti-Tray 2000® kits (IDEXX Laboratories, Westbrook, Maine). Most samples were diluted 1:50 for Colilert® and 1:10 for Enterolert® using sterile deionized water. The colony-forming units (CFU) per 100 mL were determined using the manufacturer’s most probable number (MPN) table. If the MPN measured from a sample site fell above or below the measurable range, subsequent samples collected from that site were diluted more or less, respectively. For some sample sites with low *E. coli* concentrations, the Colilert® test was conducted in two sets: one with a 1:50 dilution to quantify the total coliforms concentration and one with a 1:25 or 1:30 dilution to quantify the *E. coli* concentration.

### Isolation and classification of *E. coli*

Individual 1-mL, 5-mL, and 10-mL aliquots of stream water were filtered onto sterile 0.45-µm-pore-size membranes (Fisher Scientific, Pittsburgh, Pennsylvania). The membranes were incubated on mTEC agar (Becton Dickinson, Franklin Lakes, New Jersey) at 35 °C for 1 h and then 45 °C for 16–20 h. Yellow, yellow-green, and yellow-brown colonies growing on the mTEC plates were then streaked onto tryptic soy agar (Fisher Technical Company, Roselle, Illinois) and incubated overnight at 35 °C (United States Environmental Protection Agency (USEPA), 2002; McLellan et al., 2007). Colonies isolated from those plates were further identified as *E. coli* using the RapID Remel Spot Indole (Remel Inc., Lenexa, KS) and 4-methylumbelliferyl-β-D-glucuronide (MUG) tests (Becton Dickinson, Sparks, MD) (McLellan et al., 2007) before proceeding with triplex PCR (Clermont et al., 2000).

Triplex PCR was performed for every *E. coli* isolate following the conditions and primers (Eurofins Genomics, Louisville, KY) described by Clermont et al. (2000) using 2X GoTaq Green Mastermix (Promega, Madison, WI). The amplicons were analyzed using gel electrophoresis, and based on the presence of *chuA* (279-bp), *yjaA* (211-bp), and TspE4.C2 (152-bp), each *E. coli* isolate was classified into one of four ECOR phylogenetic groups: A, B1, B2, or D. Isolates displaying an ambiguous band pattern were omitted from the analysis, as were those with the A_0_ genotype (no bands present), since they are unlikely to truly fall within phylogroup A (Gordon et al., 2008). Thus, 736 out of 827 total isolates (89%) were assigned to an ECOR phylogroup with confidence and were used in statistical analyses.

### Statistical analyses

Statistical analyses were performed using R version 4.3.1 (the R Foundation for Statistical Computing, Vienna, Austria). We used an alpha level of 0.05 for all analyses. For sites sampled more than once during a summer, the median concentrations of total coliforms, *E. coli*, and *Enteroccocus* were used. For all other measured variables, the mean values for each site were used.

In comparisons of two independent groups (i.e., watersheds with and without PRFs in 2017 and mixed forest/pasture and forested sites in 2018), no stream sampling sites that occurred upstream of another site were included to ensure statistical independence. Therefore, one site (MD03, with no upstream PRFs) was excluded from these comparisons, resulting in sample sizes of seven for sites without upstream PRFs. Residuals were checked for normality using Shapiro-Wilk tests, and equality of variances were checked using Bartlett tests. In some cases, these assumptions were met following log_10_-transformations. If data met both assumptions, independent samples *t* tests assuming equal variances were used to compare the means of the two groups. If data met the assumption of normality but variances were unequal, Welch’s tests were used to compare the means of the two groups. Otherwise, Mann–Whitney tests were used to compare the medians of the two groups. To compare data from eight sites sampled in both 2017 and 2018, we first used Shapiro–Wilk tests to determine if the differences between the paired data were normally distributed. If the differences were normally distributed, we used paired *t* tests. Otherwise, we used Wilcoxon signed-rank tests. Calculations of Cohen’s *d* were made to estimate effects sizes for significant two-sample tests using the *lsr* package (v. 0.5.2) in R. Following the guidelines of Cohen (1988), we considered *d* values of 0.2, 0.5, and 0.8 to indicate small, medium, or large effect sizes, respectively.

We used chi-squared tests of association (independence) to determine if the frequencies of *E. coli* phylogroups differed between watershed categories or between years. If significant differences in frequencies were found, we determined which observed values deviated significantly from their corresponding expected values using post hoc analysis of adjusted residuals (Everitt, 1977; Haberman, 1973). Also, effect sizes for chi-square tests in which significant associations were found were expressed as Cramer’s *V* using the *chisquare* package (v 1.0) in R. Following the guidelines of Cohen (1988), we considered *V* values of 0.10, 0.30, and 0.50 to indicate small, medium, and large effect sizes, respectively. To test for correlations between measured variables, we used Spearman’s rank correlations given that most relationships were non-linear even after log_10_-transformations of the data.

## Results

### Comparing watersheds with and without poultry-rearing facilities

**Fig. 2:**
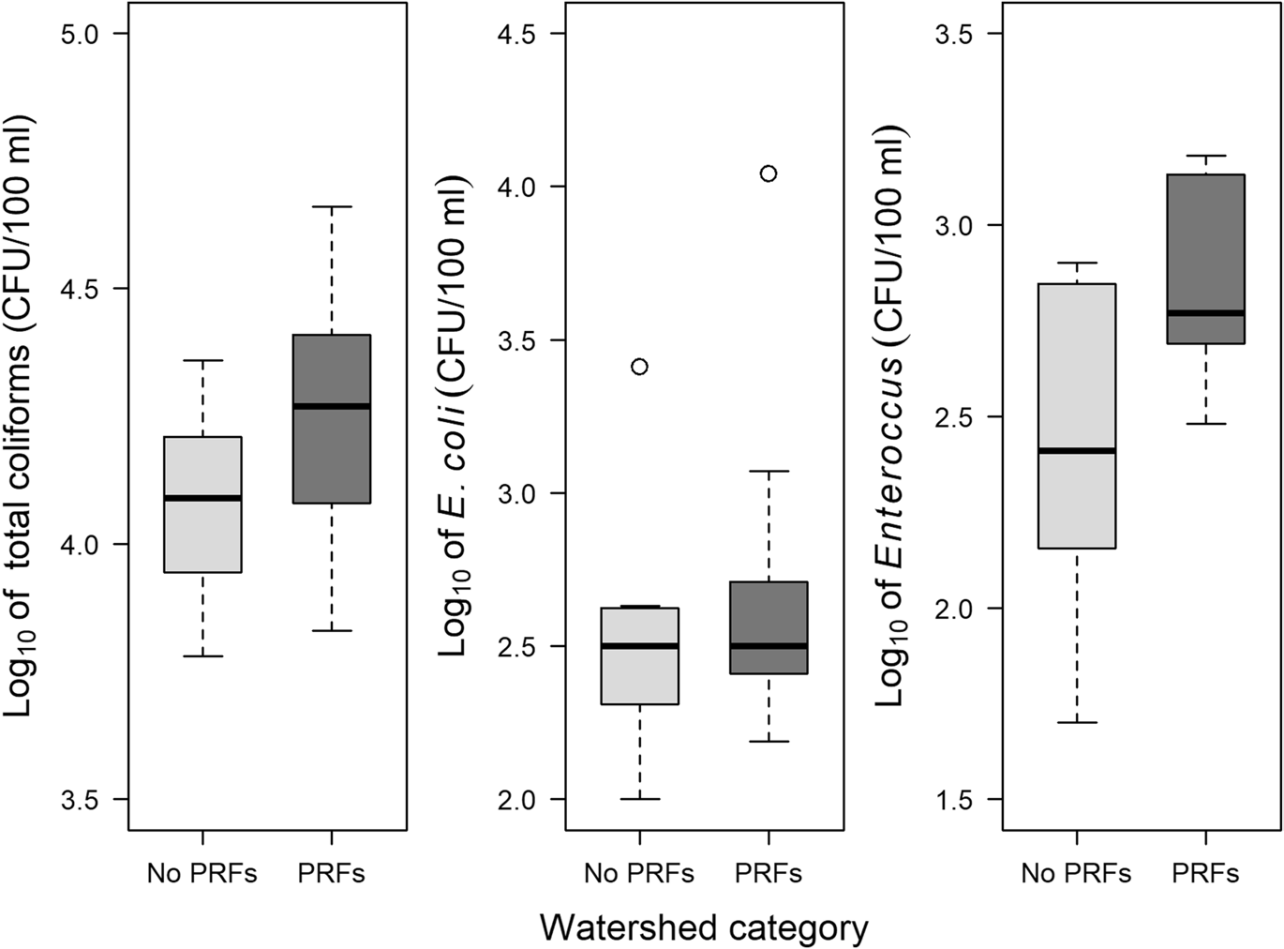
Concentrations of bacteria in streams draining rural watersheds with (*n* = 9) and without (*n* = 7) poultry rearing facilities (PRFs) in the upper Savannah River basin of South Carolina, June–July 2017. Each box represents the interquartile range, and the horizontal line within each box represents the median. Whiskers represent all values within 1.5 times the interquartile range, and circles represent outliers. Concentrations of total coliforms and *Escherichia coli* did not differ between the two categories of watersheds (total coliforms: independent samples *t* test, *t* = −1.46, df = 14, *p* = 0.17; *E. coli*: Mann–Whitney test, *W* = 26, *p* = 0.60). Concentrations of *Enterococcus* were significantly higher in watersheds with PRFs than in those without PRFs (independent samples *t* test using log_10_-transformed data, *t* = −2.29, df = 14, *p* = 0.038; Cohen’s *d* = 1.17)

In 2017, *Enterococcus* concentrations were significantly higher in watersheds with PRFs than in those without PRFs, with median concentrations being ~2.3 times higher in watersheds with PRFs (Fig. 2). Turbidity also was significantly higher in watersheds with PRFs, with the mean in watersheds with PRFs being ~1.5 times higher than in watersheds without PRFs (Table 2). However, there were no significant differences between the two groups of watersheds in concentrations of total coliforms and *E. coli* (Fig. 2) or in discharge, pH, water temperature, specific conductance, or dissolved oxygen (Table 2). Also, elevations of sample locations for the two groups spanned similar ranges (Table 2), and in neither group did concentrations of total coliforms, *E. coli*, or *Enterococcus* correlate significantly with site elevation (Spearman’s rank correlations, all *p* values ≥0.064).

**Table 2:** Physical and chemical parameters of streams sampled in rural watersheds in the Piedmont and Blue Ridge physiographic provinces of South Carolina in June–July 2017 or June–July 2018. Streams sampled in 2017 drained watersheds with or without poultry rearing facilities (PRF’s). Sample site elevations are medians with ranges. For other variables, values are either means ± SE (if data in both groups were normally distributed) or medians with interquartile ranges (if data in one or both groups were not normally distributed)

Watershed category	*n*	Site elevation (m)	Discharge (L/s)	pH	Water temp. (°C)	Specific conductance (uS/cm)	DO (mg/L)	Turbidity (NTU)
2017								
Without PRFs	7	199 (186–283)	26 (18, 31)^a^	6.49 ± 0.03^b^	22.2 (21.9, 24.7)^c^	55.5 (55.2, 57.0)^d^	6.9 ± 0.3^e^	7.8 ± 1.5^f^
With PRFs	9	237 (171–260)	26 (15, 30)	6.55 ± 0.06	22.0 (21.7, 23.5)	57.6 (54.0, 59.2)	7.0 ± 0.1	11.5 ± 0.9
2018								
Mostly forested	6	271 (211–307)	36 ± 6^g^	6.46 ± 0.16^h^	20.2 ± 0.5^i^	36.9 ± 5.3^j^	8.4 ± 0.1^k^	4.9 ± 0.8^l^
Forest/pasture	8	201 (186–283)	21 ± 5	6.45 ± 0.06	23.1 ± 1.0	52.8 ± 4.1	7.2 ± 0.3	8.6 ± 1.8

*DO* dissolved O_2_

^a^Mann–Whitney test, *W* = 28, *p* = 0.76

^b^Independent samples *t* test, *t* = −0.94, df = 14, *p* = 0.36

^c^Independent samples *t* test, *t* = 1.23, df = 14, *p* = 0.24

^d^Independent samples *t* test, *t* = −1.05, df = 14, *p* = 0.31

^e^Independent samples *t* test, *t* = −0.29, df = 14, *p* = 0.77

^f^Independent samples *t* test, *t* = −2.26, df = 14, *p* = 0.04, Cohen’s *d* = 1.14

^g^Independent samples *t* test, *t* = 2.09, df = 12, *p* = 0.059

^h^Independent samples *t* test, *t* = 0.089, df = 12, *p* = 0.93

^i^Independent samples *t* test, *t* = −2.30, df = 12, *p* = 0.040, Cohen’s *d* = 1.24

^j^Independent samples *t* test, *t* = −2.41, df = 12, *p* = 0.033, Cohen’s *d* = 1.30

^k^Independent samples *t* test (Welch’s test), *t* = 3.22, df = 9.04, *p* = 0.010, Cohen’s *d* = 1.64

^l^Independent samples *t* test (Welch’s test), *t* = −1.84, df = 9.49, *p* = 0.098

**Fig. 3:**
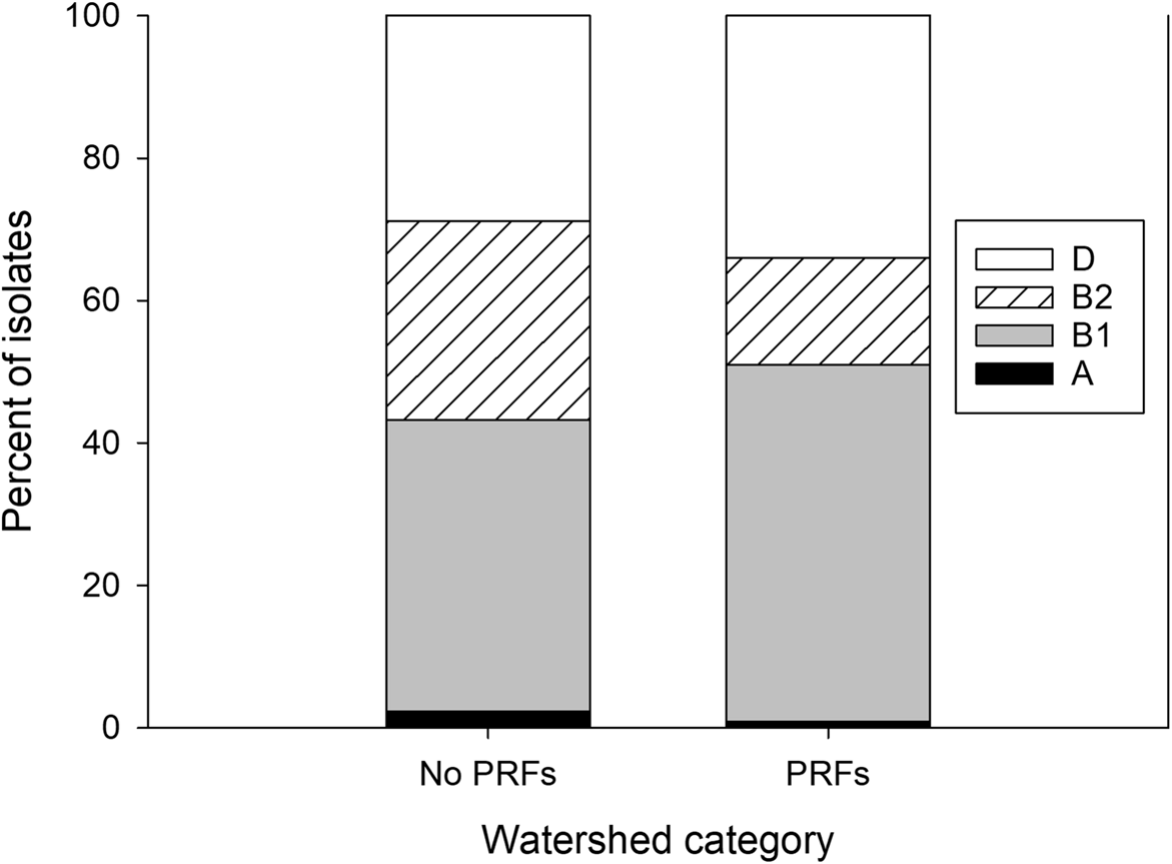
Distribution of four *Escherichia coli* phylogroups in stream water samples collected from nine streams downstream of poultry rearing facilities (“PRFs”, *n* = 239 isolates) and eight streams that were not downstream of poultry rearing facilities (“No PRFs”, *n* = 129 isolates) in the South Carolina Piedmont, June–July 2017. Frequencies of the B1, B2, and D phylogroups differed significantly between watersheds with and without PRFs (*χ*^2^ = 9.79, df = 2, *p* = 0.0075; Cramer’s *V* = 0.163)

Few isolates of *E. coli* phylogroup A were obtained from either watersheds without PRFs (three isolates) or watersheds with PRFs (two isolates) (Fig. 3). The distributions of the other three phylogroups differed significantly in streams draining watersheds with and without PRF, although the effect size was small (Fig. 3). Based on analysis of adjusted residuals, the observed frequencies of the B2 group differed significantly (*p* < 0.005) from their corresponding expected values. Specifically, B2 isolates were significantly more frequent than expected in watersheds without PRFs (observed = 37, expected = 25.6) and significantly less frequent than expected in watersheds with PRFs (observed = 36, expected = 47.4). Although the observed and expected frequencies of the B1 group did not differ significantly (*p* > 0.05), B1 isolates accounted for 50% of isolates in watersheds with PRFs in contrast to 41% in watersheds without PRFs.

### Comparing mostly forested and forest/pasture watersheds

#### Physical/chemical stream water properties and FIB concentrations

**Fig. 4:**
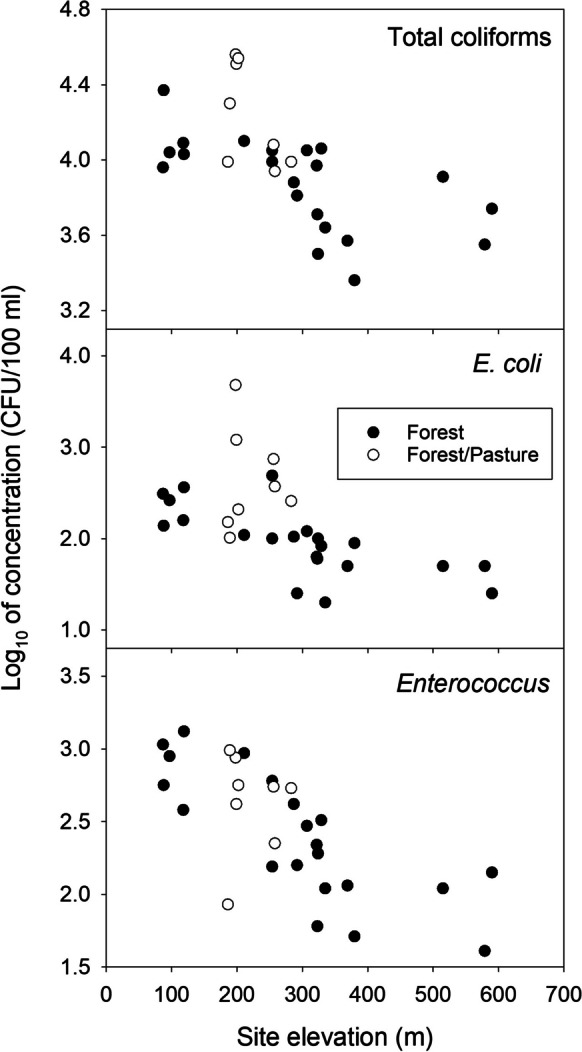
Relationships between sample site elevation and concentrations of total coliforms, *Escherichia coli*, and *Enterococcus* in 21 streams draining watersheds with primarily forest land cover (black circles) and eight streams draining watersheds with a mixture of forest and pasture land cover (white circles) in northwestern South Carolina. All samples were collected under baseflow conditions during June–July 2018. Each symbol represents either a single sample or the median of two to four samples. Relationships were significant for forest sites (Spearman rank correlations, total coliforms: rho = −0.69, *p* = 0.00059; *E. coli*: rho = −0.79, *p* < 0.0001; *Enterococcus*: rho = −0.84, *p* < 0.0001), but not for forest/pasture sites (Spearman rank correlations, total coliforms: rho = −0.19, *p* = 0.66; *E. coli*: rho = 0.29, *p* = 0.50; *Enterococcus*: rho = −0.14, *p* = 0.75)

**Fig. 5:**
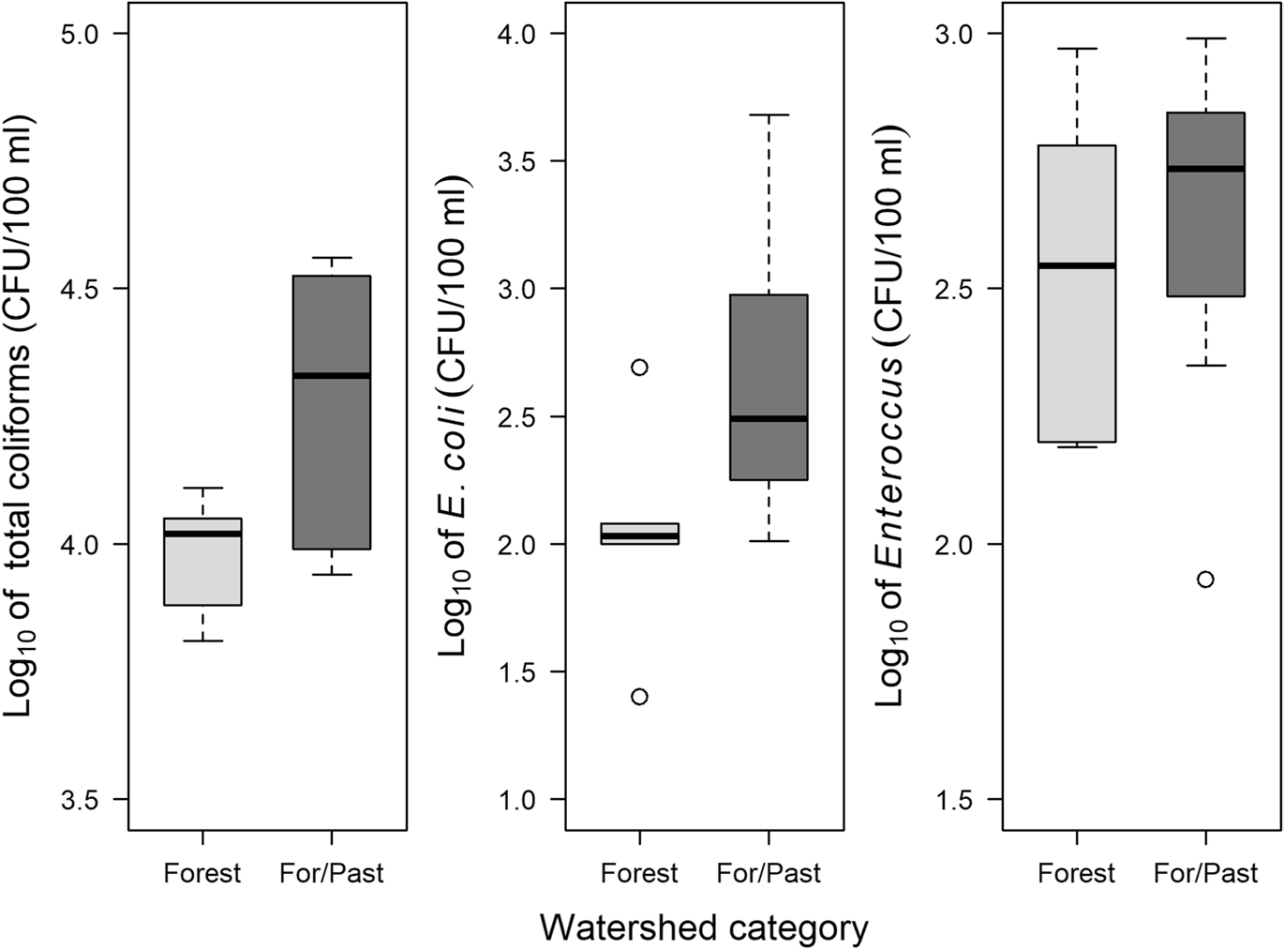
Concentrations of fecal-indicator bacteria in streams draining watersheds with primarily forest land cover (*n* = 6) and streams draining watersheds with a mixture of forest and pasture land cover (*n* = 8) in the South Carolina Piedmont, June–July 2018. Elevations of all sample collection locations ranged from 186 to 283 m. Each box represents the interquartile range, and the horizontal line within each box represents the median. Whiskers represent all values within 1.5 times the interquartile range, and circles represent outliers. Concentrations of total coliforms and *Escherichia coli* were significantly higher in the forest/pasture watersheds (total coliforms: Welch’s *t* test, *t* = −2.81, df = 7.77, *p* = 0.023, Cohen’s *d* = 1.42; *E. coli*: independent samples *t* tests using log_10_-transformed data: *t* = −2.25, df = 12, *p* = 0.044, Cohen’s *d* = 1.22). Concentrations of *Enterococcus* did not differ significantly between groups (independent samples *t* test, *t* = −0.61, df = 12, *p* = 0.55)

In 2018, the mostly forested watersheds spanned a greater range in elevation than did the forest/pasture watersheds (Table 1). Concentrations of total coliforms, *E. coli*, and *Enterococcus* were negatively correlated with sample site elevation for forest sites but not for forest/pasture sites (Fig. 4). Therefore, statistical comparisons of streams in forest/pasture and forest watersheds were conducted using only the six forest sites (BAR01, CRC03, LCC01, LCC02, POC01, US79) with sample site elevations that fell within 10% of the site elevations for the forest/pasture sites. Compared to streams in forest watersheds, streams in forest/pasture watersheds had significantly higher water temperatures, lower dissolved oxygen concentrations, and higher specific conductance (Table 2). Streams in the forest/pasture watersheds tended to have lower discharge and higher turbidity than streams in mostly forested watersheds, though these differences were not statistically significant (Table 2). Concentrations of total coliforms and *E. coli* were significantly higher in forest/pasture sites than in forest sites, with median concentrations being ~2 times and ~2.9 times higher in forest/pasture sites, respectively (Fig. 5). However, concentrations of *Enterococcus* did not differ significantly between the two groups (Fig. 5).

#### E. coli phylogroup distributions

As in samples collected in 2017, few isolates of *E. coli* phylogroup A were obtained from either the eight forest/pasture watersheds (five isolates total from all eight watersheds) or from the 21 mostly forested watersheds (two isolates total from all 21 watersheds) in 2018. Across all 21 forest sites, the percentages of *E. coli* isolates that belonged to the B1 and B2 phylogroups were correlated significantly with sample site elevation, though these relationships were only moderately strong (Supplementary Fig. 2). Specifically, the percentage of isolates that were B1 correlated negatively with site elevation, whereas the percentage of isolates that were B2 correlated positively with elevation. The percentage of isolates that were in group D did not correlate significantly with elevation (Spearman’s rank correlation, rho = −0.19, *p* = 0.43).

**Fig. 6:**
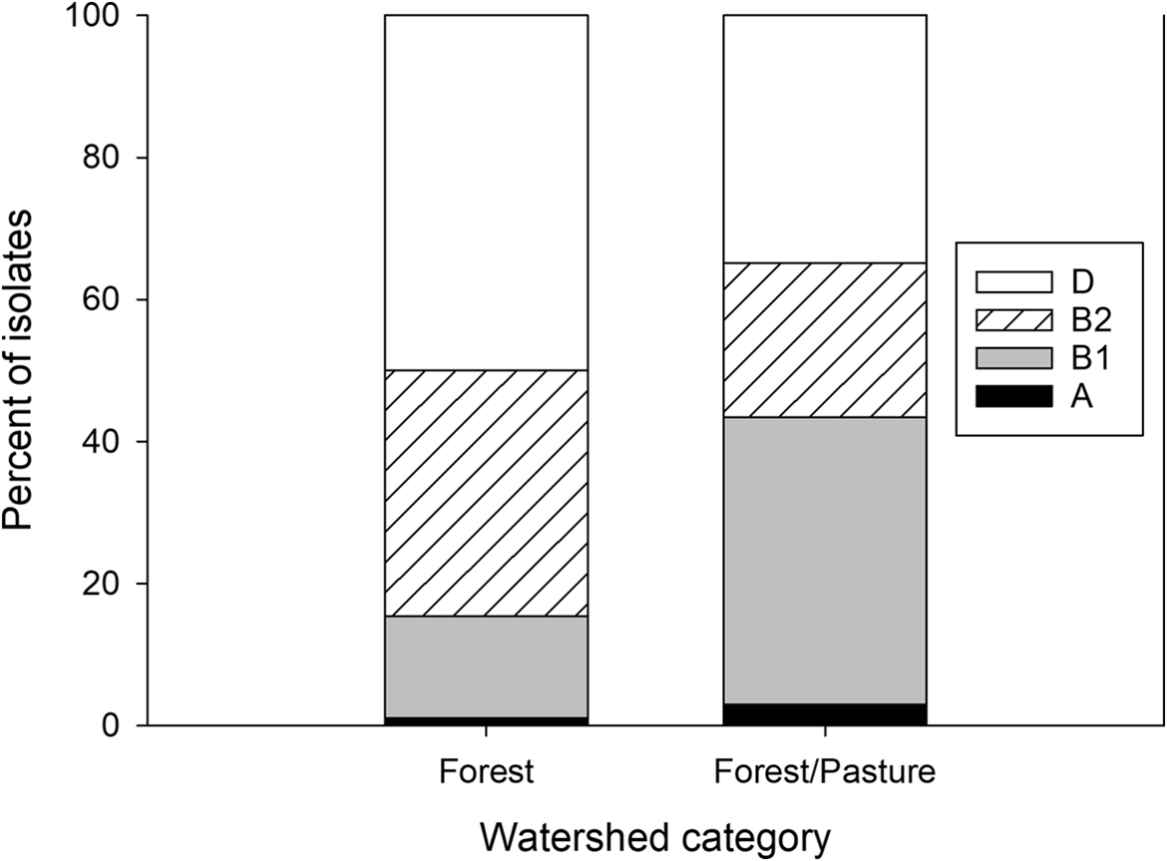
Distribution of four *Escherichia coli* phylogroups from 20 streams draining primarily forested watersheds (*n* = 182) and eight streams draining watersheds with a combination of forest and pasture land cover (*n* = 166) in northwestern South Carolina, June–July 2018. No isolates were obtained from one forest site (IR10). Frequencies of the B1, B2, and D phylogroups differed significantly between the two groups of watersheds (*χ*^2^ = 31.79, df = 2, *p* < 0.0001, Cramer’s *V* = 0.305)

We compared the distribution of phylogroups B1, B2, and D between forest and forest/pasture watersheds using all forest sites but also with only the six forest sites closest in elevation to the forest/pasture sites. Using all forest sites, the distributions of the three phylogroups differed significantly between streams in the two groups of watersheds, with a medium effect size (Fig. 6). Post hoc analysis of adjusted residuals showed significant differences between the observed and expected frequencies for phylogroups B1, B2, and D (*p* < 0.01 for all three comparisons). Specifically, B1 isolates were significantly more frequent than expected in forest/pasture watersheds (observed = 67, expected = 43.9) and significantly less frequent than expected in forest watersheds (observed = 26, expected = 49.1). In contrast, B2 isolates were significantly more frequent in forest watersheds than expected (observed = 63, expected = 52.3) and less frequent than expected in forest/pasture watersheds (observed = 36, expected = 46.7). Likewise, D isolates were significantly more frequent in forest watersheds than expected (observed = 91, expected = 78.7) and less frequent than expected in forest/pasture watersheds (observed = 58, expected = 70.3). Omission of data from forested sites with PRFs in their watersheds (KC02, with 13 isolates total, and SC01, with only one isolate; no isolates obtained from IR10) did not change these statistical conclusions.

Using only the six forest sites with elevations closest to those of the forest/pasture sites, we reached similar statistical conclusions. As in the analysis with all forest sites, the distributions of *E. coli* phylogroups B1, B2, and D differed significantly between forest and forest/pasture sites (*χ*^2^ = 19.40, df = 2, *p* < 0.0001), although the effect size was small (Cramer’s *V* = 0.289). B1 isolates were significantly (*p* < 0.01) more frequent than expected in forest/pasture watersheds (observed = 67, expected = 52.5) and significantly less frequent than expected in forest watersheds (observed = 9, expected = 23.5). Likewise, D isolates were significantly (*p* < 0.01) more frequent in forest watersheds than expected (observed = 41, expected = 30.6) and less frequent than expected in forest/pasture watersheds (observed = 58, expected = 68.4). In this comparison with fewer forest sites, observed and expected frequencies of B2 isolates did not differ significantly for either watershed group (*p* > 0.05). However, the trends were similar to the trends in the comparison using all of the forest sites, with forest sites having more B2 isolates than expected (observed = 22, expected = 17.9) and forest/pasture sites having fewer B2 isolates than expected (observed = 36, expected = 40.1).

### Interannual variation

**Fig. 7:**
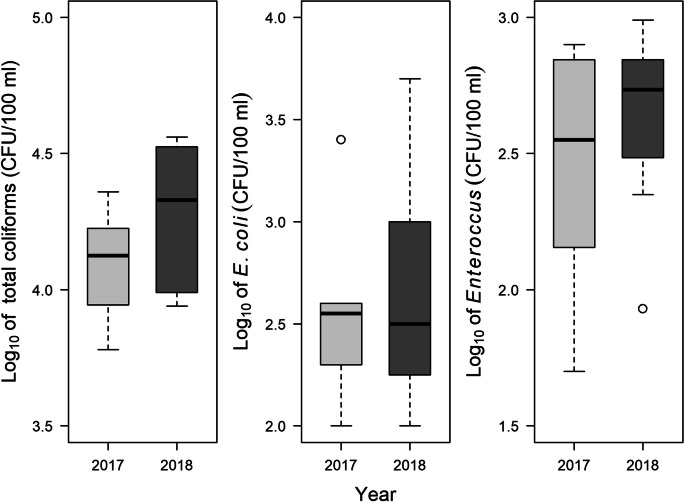
Concentrations of fecal-indicator bacteria in eight streams draining watersheds with a mixture of forest and pasture land cover sampled in both June–July 2017 and June–July 2018 in northwestern South Carolina. Each box represents the interquartile range, and the horizontal line within each box represents the median. Whiskers represent all values within 1.5 times the interquartile range, and circles represent outliers. Concentrations of total coliforms were significantly higher in 2018 than in 2017 (paired *t* test, *t* = −2.36, df = 7, *p* = 0.05, Cohen’s *d* = 0.83). Concentrations of *Escherichia coli* and *Enterococcus* did not differ significantly between years (*E. coli*: Wilcoxon signed-rank test, *V* = 9, *p* = 0.23; *Enterococcus*: paired *t* test, *t* = −0.92, df = 7, *p* = 0.39)

In streams draining watersheds with mixed forest/pasture land cover, concentrations of total coliforms were significantly higher in 2018 than in 2017, with median concentrations being ~1.6 times higher in 2018 (Fig. 7). Turbidity also was significantly higher (paired *t* test, *t* = −2.76, df = 7, *p* = 0.028) in 2018 (mean = 8.6 NTU, SE = 1.8 NTU) than in 2017 (mean = 7.1 NTU, SE = 1.5 NTU). However, there were no significant differences in concentrations of *E. coli* or *Enterococcus* between the 2 years (Fig. 7). There also were no significant differences in discharge, water temperature, pH, dissolved oxygen, or specific conductance (paired *t* tests or Wilcoxon signed-rank tests, *p* > 0.15). Finally, the frequencies of *E. coli* phylogroups B1, B2, and D did not differ significantly between the 2 years (Supplementary Fig. 3).

## Discussion

### Comparing FIB and ECOR types in watersheds with and without poultry-rearing facilities

Our results suggest that variation in the abundance of FIB and genetic diversity of *E. coli* in headwater streams in the South Carolina Piedmont are associated with both poultry production and pasture land cover, at least under summer baseflow conditions. In the case of PRFs, we found the median concentration of *Enterococcus* in streams draining watersheds with PRFs to be higher than the median concentration in streams in watersheds without PRFs (Fig. 2). However, we found no significant differences in concentrations of either total coliforms or *E. coli*. These results from stream water analyses are consistent with results from previous studies of bacteria in poultry litter and soil. For example, Brooks et al. (2009) reported that enterococci concentrations in poultry litter were three orders of magnitude higher than concentrations of total coliforms. Also, in a greenhouse-based experiment, Brooks et al. (2009) found that simulated rain runoff from soil amended with poultry litter had elevated abundance of enterococci but not of total coliforms relative to soil without litter added. Likewise, Cook et al. (2014) found that abundance of enterococci in soil was increased more than was the abundance of *E. coli* following poultry litter application.

Many factors, such as sunlight, temperature, salinity, starvation, predation by protozoans and bacteriophages, contribute to the variability of enterococci persistence in the environment (Byappanahalli et al., 2012). Although it was suggested decades ago that the enterococci might be a more reliable FIB than *E. coli* (Ostrolenk et al., 1947) and currently they are the only FIB recommended by the United States Environmental Protection Agency for brackish and marine water (United States Environmental Protection Agency (USEPA), 2004), their applicability in assessing freshwater quality could be challenging. Fecal coliforms have greater persistence than enterococci in freshwater (Anderson et al., 2005). However, being Gram-positive, enterococci are more resistant to protozoan grazing (Iriberri et al., 1994) and abiotic environmental stressors (Bale et al., 1993), and they could become endogenous in soil (Byappanahalli & Fujioka, 2004). Although Byappanahalli et al. (2012) suggest that enterococci may be less reliable FIB than total coliforms and *E. coli*, our study suggests an association between the abundance of enterococci and PRFs.

Pasture was a major land cover in all watersheds, both those with and without PRFs. Cattle, the predominant mammalian livestock on pasture in our study area, were present in both groups of watersheds (the “with PRFs” group and the “without PRFs” group) and potentially could have been sources of fecal bacteria in the streams we sampled. However, in contrast to poultry, cattle may be greater sources of *E. coli* than of enterococci. For example, both Sinton et al. (2007) and Moriarty et al. (2008) found the initial abundance of *E. coli* in fresh cattle feces on New Zealand pastures to be up to four orders of magnitude higher than the abundance of enterococci. Thus, it seems plausible that in our study area poultry could have been a more important source of enterococci in stream water than were cattle.

Although we did not find significant differences in total *E. coli* concentrations between PRF and non-PRF sites, we did find evidence of differences in proportions of ECOR phylogroups. The relative rarity of phylogroup A in stream water in our study is consistent with a stream draining agricultural land in western Kentucky under both low and high flow conditions (Cook et al., 2011) and with the temporal characterization of *E. coli* phylotypes in the Upper Oconee watershed in northeast Georgia (Cho et al., 2018). However, we found that *E. coli* populations downstream of PRFs had lower proportions of group B2 relative to the populations that were not downstream of PRFs. Escobar-Páramo et al. (2006) found that B2 isolates were more common in wild birds than in domestic birds (mainly chickens but also turkeys and geese) in France. In our study, group B1 was the most abundant in stream water, supporting a large body of evidence that group B1 is most commonly isolated from soil (Bergholz et al., 2011) and surface water (Berthe et al., 2013; Tymensen et al., 2015) and is the group most capable of persisting outside of a host (Berthe et al., 2013; Dusek et al., 2018; Tymensen et al., 2015). Also, our data suggest that group B1 isolates were more frequent in watersheds with PRFs in contrast to watersheds without PRFs. Additionally, some previous studies have linked *E. coli* groups A and B1 to poultry (Coura et al., 2017; Hiki et al., 2014; Pasquali et al., 2015). In our study, although groups B1 and D were both prevalent in populations downstream of PRFs and ones without PRFs, the populations downstream of PRFs had a slightly higher proportion of groups B1 and D. The influence of PRFs on stream water *E. coli* phylogroups could also depend on the waste management practices of the facility. Applying poultry litter to agricultural or pasture land may be a common practice in the study region (Camberato, 2007), but application timing, extent of composting, and levels of precipitation strongly impact the amount of FIB transported in runoff water (Chaubey et al., 2010; Harmel et al., 2013; Jenkins et al., 2006; Wood et al., 2012). Additional research is needed to determine the extent to which stream water *E. coli* phylogroups under baseflow conditions, without active runoff inputs, reflect endemic strains relative to poultry-associated strains (Jang et al., 2017). Furthermore, a more detailed *E. coli* phylogrouping using the revised Clermont typing scheme (Clermont et al., 2013) could be implemented to reduce the number of isolates unclassified by the traditional method (Clermont et al., 2000). Also, although we did not focus on fecal bacteria source tracking in this study, having more information about the phylogroup diversity of *E. coli* in animals (Lagerstrom & Hadly, 2023) will guide our future research directions.

### Comparing FIB and ECOR types in forest and forest/pasture

In comparing FIB abundance in mostly forested watersheds to forest/pasture watersheds, we found significantly higher total coliform and *E. coli* abundance, but not significantly higher *Enterococcus* abundance, in the forest/pasture watersheds (Fig. 5). Poultry rearing facilities were absent from both the forest/pasture watersheds and the mostly forested watersheds that occurred at similar elevations. However, cattle or other mammalian livestock were present in pasture in the forest/pasture watersheds and may have contributed to the elevated coliform abundance in the streams.

In comparing forest and forest/pasture environments, group B1 was more common in mixed forest/pasture watersheds, and group B2 was more common in the forested watersheds (Fig. 6). This result is consistent with previous studies in which the dominant phylogroup in cattle feces was group B1 (Coura et al., 2015; Howard et al., 2017; NandaKafle et al., 2017; Unno et al., 2009; Tymensen et al., 2015). Groups B2 and D were proportionally more common in forested watersheds than in mixed forest/pasture watersheds. One previous study showed that when comparing birds (both wild and domestic) and mammals, a higher prevalence of groups B2 and D were found in birds and a higher prevalence of groups A and B1 in mammals (Escobar-Páramo et al., 2006). Additionally, groups A and B1 were more likely to be associated with non-human mammals and group B2 with wild birds (Escobar-Páramo et al., 2006). Although we do not know the density of livestock or of wildlife within the mixed forest/pasture watersheds we studied, we can reasonably assume that livestock were either absent or occurred at very low densities in the mostly forested watersheds.

Our results from streams in mostly forested watersheds suggest that variation in watershed elevation influences both abundance of suspended bacteria and the frequencies of some *E. coli* phylotypes in the South Carolina Piedmont. The inverse relationships between bacterial concentrations and sample site elevation we have reported are consistent with patterns in abundance of fecal bacteria in headwater streams across an elevation gradient in the Great Smoky Mountains in North Carolina and Tennessee (Silsbee & Larson, 1982). Even though the range of elevation in the Smoky Mountains was several hundred meters greater than in our study, bacterial abundance shows similar patterns across elevations in both studies. The causes of these relationships between bacterial abundance and elevation are unclear, but both water temperature and stream discharge may be important factors. For example, Silsbee and Larson (1982) reported that, across the elevation gradient in the Smoky Mountains, bacterial concentrations were more strongly correlated with water temperature than with other physical or chemical measurements. In summer months in the Smoky Mountains, stream water temperature was inversely related to mean basin elevation (Silsbee & Larson, 1982). We found a similar pattern in that stream water temperatures in the forested watersheds we studied in summer months were lowest at the highest elevation sites (17 to 18 °C) and highest (23 to 26 °C) at the lowest elevation sites. Thus, higher bacterial concentrations in the lower elevation streams were associated with warmer water temperatures. Previous studies in other regions have provided experimental evidence that increasing water temperature can increase production rates of bacteria in both stream sediments (Sand-Jensen et al., 2007) and surface water (Adams et al., 2010). Whether temperature influences the abundance of suspended fecal indicator bacteria directly or indirectly in streams in our region is unknown and requires further study.

Also, in our study, stream baseflow discharge in forest streams varied markedly across the elevation gradient, from 0.1–2 L/s at the lowest elevation streams to 80–290 L/s at the highest elevation streams. Thus, bacterial abundance was inversely related to steam discharge. Silsbee and Larson (1982) likewise note that in the Great Smoky Mountains, streams at higher elevations have higher average discharge compared to streams at lower elevations. These baseflow discharge patterns, which are consistent with comparisons of Appalachian Mountain and Piedmont watersheds in previous studies (e.g., Zimmer & Gannon, 2018), suggest higher groundwater inputs to streams at higher elevations than at lower elevations. Groundwater inputs could also relate to concentrations of bacteria in stream surface waters. For example, Rochelle-Newall et al. (2016) suggest that *E. coli* concentrations in stream or river water could be diluted by groundwater inputs where *E. coli* concentrations in groundwater are low and groundwater inputs are high relative to surface water flow. As with the influence of water temperature on bacterial abundance, the degree to which stream discharge and groundwater inputs influence FIB concentrations in our region is uncertain and requires further study.

### Comparing FIB and ECOR types in 2017 and 2018

Several studies have reported seasonal differences in FIB abundance and diversity in stream water, perhaps based on changing temperatures, precipitation, and wildlife migration patterns (e.g., Badgley et al., 2019; Chandran & Mazumder, 2015; Cho et al., 2018; Jang et al., 2014). Fewer studies have assessed the temporal variation of FIB populations in stream water on an interannual scale. Under summer baseflow conditions, we found no difference in *E. coli* abundance or genetic diversity in mixed forest/pasture watersheds between two consecutive years. Stream physical and chemical conditions, such as water temperature, discharge, pH, dissolved oxygen, and specific conductance were similar at the time of samplings in 2017 and 2018. It would be useful for future studies to examine whether the relatively stable genetic diversity of ECOR phylotypes relates to the ability of *E. coli* to persist in the environment and whether specific biotic or abiotic factors select for particular strains within those streams.

### Study limitations and future research directions

Although *E. coli* thrives as a commensal gut microbe, it is estimated that half of the *E. coli* exist outside of a host species (Savageau, 1983). Because *E. coli* is capable of surviving, replicating, and evolving in the extrahost environment, many researchers have called into question the usefulness of *E. coli* as an indicator of fecal contamination (Brennan et al., 2010; Ishii et al., 2006; Jang et al., 2017; Somorin et al., 2016; Stocker et al., 2015; Van Elsas et al., 2011; Walk et al., 2007). Nevertheless, our study suggests that livestock production may influence the abundance and genetic diversity of *E. coli* in surface waters. To further associate animal sources of contamination in water quality, we will investigate members of the cryptic lineages of *E. coli* clade I to V in the future because some of the environmentally naturalized members of the clades are found to be abundant in non-human mammals and in birds (Clermont et al., 2011; Walk et al., 2009). More research also is needed to understand how environmental factors influence the abundance and genetic diversity of both transient and naturalized *E. coli* populations in streams and other surface waters.

Finally, we acknowledge that we only collected samples in summer months, yet concentrations of suspended bacteria can vary seasonally in streams and rivers. For example, in forest streams in the Great Smoky Mountains, Silsbee and Larson (1982) reported that concentrations of coliforms and fecal streptococci were highest in summer and lowest in winter within a given range of watershed elevations. Stocker et al. (2019) report higher concentrations of *E. coli* and enterococci in summer than in winter in water, sediment, and on periphyton in a stream in the Maryland coastal plain. It would be useful for future studies to examine whether the frequencies of *E. coli* phylotypes in stream water vary seasonally, as well. In particular, it would be useful to determine, for watersheds with livestock production, whether the distribution of in-stream phylotypes more closely reflects the phylotypes found predominantly in livestock during seasons in which there is greater overland flow due to higher precipitation and/or more saturated soils.

## Conclusions

To our knowledge, our study is the first to examine whether the distribution of *E. coli* phylogroups in small streams varies between watersheds with and without animal production systems. In particular, *E. coli* isolates of the B1 phylogroup were more frequent and B2 isolates were less frequent in watersheds with poultry rearing facilities than in those without. We also found evidence that streams draining watersheds with poultry farms had higher *Enterococcus* concentrations compared to streams draining watersheds without poultry farms. Additionally, our study suggests that pasture land cover may influence the concentrations of fecal indicator bacteria and the genetic diversity of *E. coli* in stream water. Streams in watersheds with mixed forest and pasture land covers had significantly higher concentrations of total coliforms and *E. coli* than streams in mostly forested watersheds. Streams draining mixed forest/pasture watersheds also had higher frequencies of phylogroup B1 *E. coli* isolates than streams in mostly forested watersheds. Overall, *E. coli* populations in streams draining watersheds with poultry farms and in streams draining forest/pasture watersheds had proportionally more of the ECOR phylogroups associated with livestock. By contrast, *E. coli* populations in streams draining primarily forested watersheds had a higher proportion of ECOR phylogroups that have been associated in previous studies with wild animal sources than with livestock. We also found that frequencies of the different *E. coli* phylogroups were consistent in two consecutive summers streams draining mixed forest/pasture watersheds.

We also found in streams draining mostly forested watersheds that concentrations of total coliforms, *E. coli*, and *Enterococcus* varied inversely with sample site elevation. Along this elevation gradient (from lower elevations in the Piedmont province to higher elevations in mountainous terrain in the Blue Ridge province), bacterial concentrations were highest in lower elevation streams where water temperatures were highest and stream discharge was lowest. Such elevational variation could confound efforts to determine the relationship between bacterial concentrations and livestock production unless watersheds of similar elevations are compared as in our study. In particular, there may be limited availability of watersheds with minimal development (e.g., mostly forested land cover) for comparison to watersheds with high levels of land cover alteration or land use intensity. In our study, we could find few mostly forested watersheds that occurred at elevations similar to those of mixed forest/pasture watersheds. In the South Carolina Piedmont, watersheds covered mostly by forest are rare outside of protected lands, such as state parks or state and national forests. Similar challenges to locating forested watersheds for comparison to more anthropogenically altered watersheds are likely to occur in other geographic regions. Further research is needed to understand how changing physical and chemical conditions of streams along elevation gradients influence bacterial abundance and diversity.

## Supplementary Information

Below is the link to the electronic supplementary material.

Supplementary file1 (DOCX 18.0 MB)

## Data Availability

No datasets were generated or analysed during the current study.
