# Supplementary material for: Abundance of fecal indicator bacteria and diversity of Escherichia coli associated with poultry farms and pasture land cover in streams of northwestern South Carolina
Source: Environ Monit Assess. 2024 Dec 4;197(1):15. doi: 10.1007/s10661-024-13499-w (PMC11618215; doi:10.1007/s10661-024-13499-w)
Supplement: Supplementary file 1 — Supplementary file1 (DOCX 18.0 MB) [file 10661_2024_13499_MOESM1_ESM.docx]

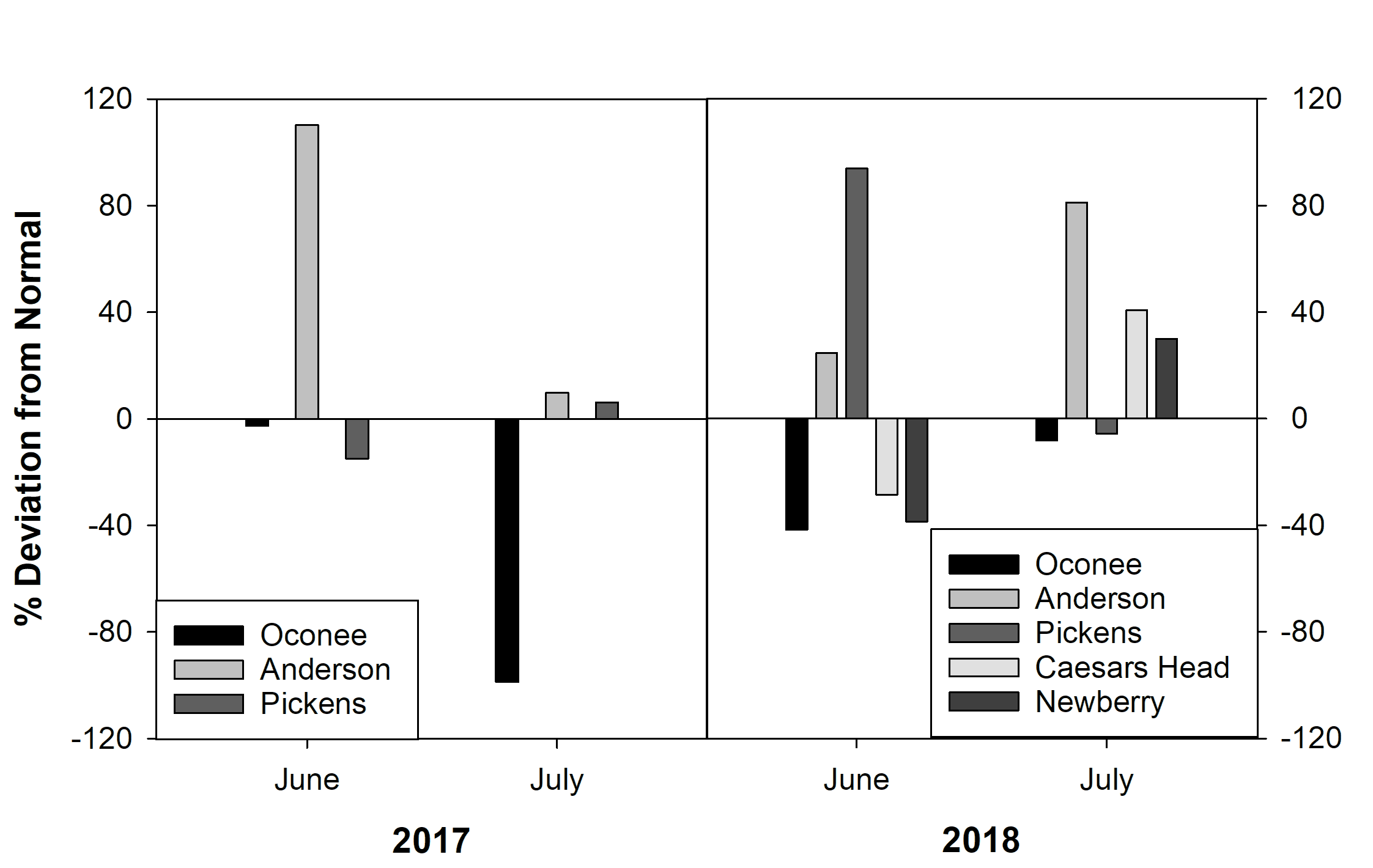


**Supplementary Fig. 1** Deviation from normal in monthly precipitation at five climate monitoring stations in the South Carolina Piedmont (Oconee, Anderson, Pickens, and Newberry) and Blue Ridge (Caesars Head) Physiographic provinces. Stations are the same as those shown in Fig. 1. Data were obtained from the U. S. National Oceanic and Atmospheric Administration’s National Centers for Environmental Information (https://www.ncdc.noaa.gov/)





**Supplementary Fig. 2** Percentages of *Escherichia coli* isolates that belonged to the B1 or B2 phylogroups in 20 streams draining mostly forested watersheds in northwestern South Carolina, June-July 2018. Each symbol represents a single stream sampling site. Isolates from each stream were classified as belonging to the A, B1, B2, or D phylogroups. For both the B1 and B2 phylogroups, the relationships with sample site elevation were significant (Spearman rank correlations: B1, rho=-0.49, p=0.03; B2, rho=0.58, p=0.007)

**

**

**Supplementary Fig. 3** Distribution of four *Escherichia coli* phylogroups from 8 streams draining watersheds with a mixture of forest and pasture land cover sampled in both June-July 2017 (n=143 isolates) and June-July 2018 (n=166 isolates) in northwestern South Carolina. Frequencies of the B1, B2, and D phylogroups did not differ significantly between years (χ^2^=2.52, df=2, p=0.28)
